# Effect of diatomite and KMnO_4_-impregnated diatomite in LDPE films on ethylene removal and postharvest quality of bananas

**DOI:** 10.1016/j.fochx.2026.104499

**Published:** 2026-09-26

**Authors:** Theara Yann, Charinee Winotapun, Phanny Yos, Lee Hwei Voon, Yew Hoong Wong, Panita Ngamchuachit, Orathai Boondamnoen

**Affiliations:** a Department of Materials Science, Faculty of Science, Chulalongkorn University, Bangkok 10330, Thailand; b PTT MCC BIOCHEM Co., Ltd., 555/1 Energy Complex Tower, Building A 16th Floor, Vibhavadi Rangsit Road, Chatuchak, Bangkok 10900, Thailand; c Research and Innovation Center, Institute of Technology of Cambodia, Russian Federation Blvd., P.O. Box 86, Phnom Penh 12156, Cambodia; d Nanotechnology and Catalysis Research Centre, Universiti Malaya, 50603 Kuala Lumpur, Malaysia; e Department of Mechanical Engineering, Faculty of Engineering, Universiti Malaya, Kuala Lumpur 50603, Malaysia; f Centre of Advanced Materials, Faculty of Engineering, Universiti Malaya, Kuala Lumpur 50603, Malaysia; g Department of Food Technology, Faculty of Science, Chulalongkorn University, Bangkok 10330, Thailand; h Current address: Singapore Institute of Food and Biotechnology Innovation (SIFBI), Agency for Science, Technology, and Research (A*STAR), 31 Biopolis Way, Nanos, Singapore 138669, Singapore; i Center of Excellence in Upcycled Materials from Industrial and Agricultural Wastes; Department of Materials Science, Faculty of Science, Chulalongkorn University, Phyathai Road, Bangkok, 10330, Thailand

**Keywords:** Alkaline activated diatomite, Ethylene scavenger ethylene removal capacity, Potassium permanganate impregnated diatomite, Thermal properties, Mechanical properties, Shelf life, Banana

## Abstract

This study developed ethylene-scavenging films by incorporating potassium permanganate (KMnO_4_)-impregnated diatomite into a low-density polyethylene (LDPE) matrix. Various additives, including raw, alkaline-activated, and KMnO_4_-impregnated diatomite (DA-KMnO_4_ and Alk-DA-KMnO_4_), were incorporated into the films and tested. The additives did not improve mechanical or gas-barrier properties; however, they significantly enhanced thermal stability, crystallinity, and ethylene-removal capacity. The highest ethylene-removal capacities were achieved by the 1% DA/LDPE and 1% Alk-DA-KMnO_4_/LDPE films, which exhibited capacities of 296.58 ± 2.07 μL/g, and 373.22 ± 13.83 μL/g, respectively. Practical storage trials using bananas, a climacteric fruit, demonstrated the efficacy of films. Bananas stored in neat LDPE ripened within 6 days, whereas those in 1% DA/LDPE films stayed green and fresh for up to 10 days, while those in 1% Alk-DA-KMnO_4_/LDPE turned green-yellow after 9 days. Ultimately, these KMnO_4_-based diatomite-LDPE films offer a viable solution for delaying ripening, maintaining firmness, and extending the shelf life of fresh produce.

## Highlights

• Low Density Polyethylene (LDPE) films containing KMnO₄-impregnated diatomite were developed for ethylene scavenging.
• The 1% Alk-DA-KMnO_4_/LDPE film achieved the high ethylene removal of 373.22 ± 13.83 μL/g.
• 1% DA/LDPE packaging extended the shelf life of green bananas up to 10 days.
• The films prevent fruit firmness and slower increase in Total Soluble Solid (TSS).

## Graphical abstract

**Figure:**
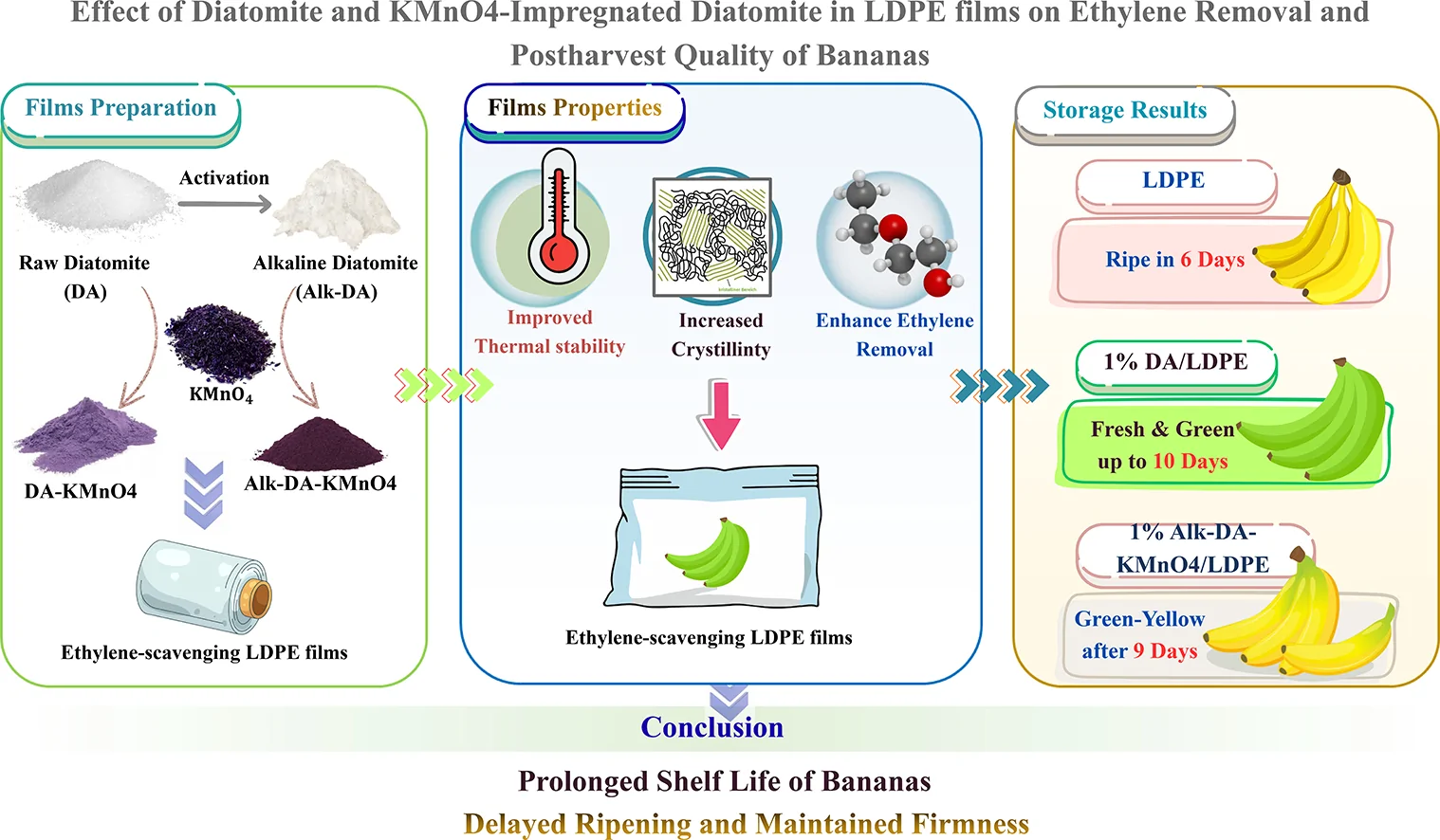

## 1 Introduction

Ethylene (C_2_H_4_) is a phytohormone gas which is released from fresh fruits and vegetable after harvest. The presence of ethylene gas has effects respiration on rate, color change, chlorophyll degradation, and flavor development consequently, accelerating ripening and senescence of fresh produce, which negatively influences the quality and shelf-life of fruits and vegetables (Awalgaonkar et al., 2020; Gaikwad et al., 2019). Even at very low concentrations (10–100 nL/L), ethylene significantly affects the postharvest quality of fruits and vegetables by accelerating chemical changes such as cell wall polysaccharide breakdown, chlorophyll degradation, and nutrient loss, while also inducing physical alterations including softening, texture loss, and surface browning or scalding (Sadeghi et al., 2019). Reducing the amount of ethylene gas surrounding fresh produce is crucial to mitigate postharvest losses and ensure longer shelf-life and high quality of products.

Ethylene scavengers are employed to manage and reduce ethylene levels in the form of sachets. Potassium permanganate is among the most effective ethylene eliminators compared with other scavengers. However, it cannot be used directly with products due to its toxicity. For safety and practicality, potassium permanganate is incorporated into materials such as activated carbon, silica gel, activated alumina metal oxides, clay, zeolite, and layer silicates (Hernández et al., 2019; Joung et al., 2021). Wang and Ajji (2022a) improved the ethylene scavenging capacity for preserving the shelf life of avocados by mixing pumice nanoparticles of various sizes with potassium permanganate. They achieved an ethylene adsorption capacity of about 620 μL/g within 24 h at a relative humidity of 30% by using 1 g of pumice particles smaller than 5 μm combined with 100 mg of potassium permanganate. This approach can extend the shelf life of avocados by an additional week. Syamsu et al. (2016) found that 3 g of nano Zeolite-KMnO_4_ activated with KOH could remove ethylene gas at a rate of approximately 113 ppm/g. Additionally, 1 g of nano Zeolite-KMnO_4_ was effective in prolonging the shelf life of 1 kg of Ambon bananas to 23 days at 25 °C and 85% relative humidity.

Recently, ethylene scavenger compounds have been incorporated into packaging materials by melting or coating (Mariah et al., 2022). A wide range of polymers and combinations are available for food packaging, providing manufacturers with the flexibility to select the most appropriate solution for each product. Polymeric films with thicknesses ranging from 10 to 60 μm, including polyethylene (PE), polyethylene terephthalate (PET), polypropylene (PP), and polyvinyl chloride (PVC), have a high-water vapor transmission rate (WVTR) compared to fresh produce. Polyethylene is a popular packaging material due to its softness, flexibility, clarity, tear and moisture resistance. However, it is not an effective gas barrier. To overcome this limitation, inorganic nanofillers like clay and zeolite can be composited into polymer matrices to improve the barrier properties. This strategy aims to reduce or prevent the concentration of gases like ethylene, oxygen, and carbon dioxide (Mariah et al., 2022). In addition, researchers found that adding potassium permanganate (KMnO_4_) impregnated with nano clay or Halloysite nanotubes into LDPE can improve ethylene removal efficiency but it did not always improve gas barrier properties (Ebrahimi et al., 2021; Joung et al., 2021; Mariah et al., 2022). However, Boonsiriwit et al. (2020) treated halloysite nanotubes with alkaline before impregnation with KMnO_4_ was also suggested to improve ethylene removal efficiency, and gas barrier. Khosravi et al. (2013) created composite films by combining nano‑potassium permanganate (KMnO_4_) with low-density polyethylene (LDPE) in varying ratios. Their study found that although the crystallinity of the LDPE composited films decreased, the gas barrier properties improved with increasing KMnO₄ content. This improvement is attributed to KMnO₄ particles creating a more tortuous diffusion path and a more compact structural arrangement. Specifically, the addition of 5 wt% KMnO₄ led to a 22% reduction in the oxygen transmission rate compared to neat LDPE films. Boonsiriwit et al. (2020) developed a nanocomposite film by incorporating alkaline-halloysite nanotubes (ALK-HNTs) into low-density polyethylene (LDPE), significantly enhancing ethylene adsorption capacity to 5.27 ± 0.65 μL ethylene/100 cm^2^, compared to only 0.024 μL for neat LDPE. While the gas barrier and crystallinity improved with 1% ALK-HNTs, there were slight decreases in tensile strength and elongation. Packaging fresh cherry tomatoes in 3% ALK-HNTs/LDPE film effectively reduced firmness loss and weight loss at 8 °C and 90% RH, with the firmness of tomatoes in neat LDPE being 17% softer and weight loss at 21 days being 0.97% for ALK-HNTs/LDPE compared to 1.53% for neat LDPE. However, higher ALK-HNTs loading negatively affected the mechanical properties of films. Joung et al. (2021) found that loading KMnO_4_-impregnated halloysite nanotubes (HNTs) into LDPE increased crystallinity but did not optimize the gas barrier properties. Ethylene removal efficiency improved with higher concentrations, reaching 42.00 ± 1.65 μL/100 cm^2^ compared to 0.024 μL for neat LDPE. Using 1% KMnO_4_-HNTs/LDPE extended the shelf life of cherry tomatoes to 21 days at 20 °C and 80% RH, with color changes occurring more slowly than in neat LDPE.

Diatomite is an interesting material made of amorphous silica. Compared to other natural adsorbents like clays and zeolites, diatomite offers good low-cost benefits, large-scale environmental application, with unique physical and chemical properties such as extremely porous (approximately 80–90% voids), chemical inertia, poor thermal conductivity, tiny pore diameters, a large specific surface area, and a high potential for adsorption. In general, diatomite comprises 87–91% silicon oxide (SiO_2_), as well as a substantial amount of alumina (Al_2_O_3_) and iron oxide (Fe_2_O_3_). It is commonly used as a filter, adsorbent material, catalyst supporter, and filler additive in polymer resins. Its surface comprises silicon hydroxyl (Si-OH) groups, allowing it to attract organic molecules. However, the quantity of Si-OH groups on natural diatomite surfaces is often insufficient to increase this attraction. Therefore, chemical and physical activation procedures, such as acid and alkaline treatments, are often used to boost the affinity of diatomite for organic molecules (Sinpichai et al., 2023; Xue et al., 2022). Alkaline activation enhances the surface area of diatomite by dissolving impurities that clog natural pores and trap hydroxyl ions, which otherwise compromise diatomite performance. Beyond residue removal, this treatment induces partial dissolution of structural ions, prompting structural rearrangements and shifts in crystallinity. Consequently, it optimizes the composition, density of surface functional groups, specific surface area, and pore distribution, and adsorption capacity (Ettahiri et al., 2025; Tassadit et al., 2016). However, the utilization of diatomite as an ethylene adsorbent embedded within a polymer matrix to develop active scavenger films for fresh fruit packaging has not yet been investigated.

This study aimed to explore novel adsorbent materials by developing LDPE-based composite films incorporating raw diatomite, potassium permanganate (KMnO_4_), and alkaline-activated, KMnO_4_-impregnated diatomite via blown film extrusion. It was hypothesized that alkaline activation would enhance the ethylene-removal performance of diatomite and that KMnO₄ impregnation would further improve the performance. It was further hypothesized that incorporating these materials at low concentrations into LDPE would provide ethylene removal activity while retaining film properties, and would contribute to retaining banana quality during storage. Based on these hypotheses, the composite films were characterized in terms of surface morphology, thermal stability, mechanical strength, optical clarity, gas barrier performance, and ethylene scavenging capacity. The selected films were evaluated for their ability to extend the shelf life of bananas by monitoring postharvest quality parameters, including color change, firmness, weight loss, and total soluble solids (TSS).

## 2 Materials and methods

### 2.1 Materials

#### 2.1.1 Chemicals

Diatomite (Diatomacius earth) medical grade was purchased from TTK Science Co., Ltd. NaOH (Micropearls, AR1325) was purchased from RCI Labscan Limited, Thailand. KMnO_4_ (Grade AR, KemAus™, KA414) was purchased from S.M.Chemical Supplies Co.,Ltd. Low density Polyethylene (LDPE, InnoPlus LD2426K) was supplied by PPT Global Chemical Public Company Limited, Thailand. Pure ethylene gas (99.9%) was supplied by LSS Lab Science and Service Co., Ltd.

#### 2.1.2 Banana fruit

Banana fruits (*Musa acuminata*) at the mature green stage were harvested from a commercial orchard in Ratchaburi Province, Thailand, and transported to the laboratory within 4 h after harvest. Banana fruits of uniform size (Length: 20.26 ± 0.93 cm, width: 4.38 ± 0.21 cm), weight (217.87 ± 10.56 g), and peel color (L^⁎^ = 56.73 ± 0.30°, a^⁎^ = −16.16 ± 0.28°, b^⁎^ = 26.96 ± 0.50°, and Hue angle = 122.23 ± 0.04°), free from bruises or defects, were selected and randomly assigned to each treatment group for the evaluation of postharvest quality.

### 2.2 Experiment set up

#### 2.2.1 Preparation alkaline activated diatomite (Alk-DA)

Diatomite (DA) was milled and passed through a 200-mesh sieve, after which it was purified using distilled water. Subsequently, the purified diatomite powder was activated with NaOH. To summarize, ten grams of purified particle were immersed in a 100 mL of NaOH (5.0 mol L^−1^) using a magnetic stirrer at a speed of 500 rpm and heated up at 85 °C for 1 h. Subsequently, centrifugation was performed at 4000 rpm for 30 min to separate the alkaline diatomite. The samples were then washed repeatedly with distilled water until a neutral pH was achieved. Finally, samples were dried in a hot air oven at 80 °C for 24 h.

#### 2.2.2 Preparation potassium permanganate-impregnated diatomite (Alk-DA-P)

The 10 g of Alk-DA was immersed in 100 mL of 2 N (6.4 g/100 mL) KMnO_4_ and shook for 2 min. After that it was kept at a low temperature (< 20 °C) for 2 h. Then, centrifuged at 4000 rpm for 45 min to separate the supernatant from the particles. Finally, the particles were dried in the oven at 50 °C for 48 h.

#### 2.2.3 Films preparation

Prior to the film processing, a 10 wt% masterbatch was prepared. The DA, Alk-DA, neat KMnO_4_, and KMnO_4_-impregnated diatomite (DA-KMnO_4_), KMnO_4_-impregnated Alk-DA (Alk-DA-KMnO_4_) particles and LDPE resin granules were physically mixed at a ratio of 10:90 (*w*/w), and then was fed into a co-rotating twin screw extruder (Thermo Scientific Process 11, 11 mm screw diameter and ratio of screw length to diameter (L/D) of 40), and processing temperature 120 °C, 140 °C, 160 °C, 180 °C for the feed, transfer, melt, and die zones, respectively. The masterbatches were extruded through a 3 mm die diameter and cut into pellets. Subsequently, the master batch pellets of DA/LDPE, Alk-DA/LDPE, KMnO_4_/LDPE, and DA-KMnO_4_/LDPE, Alk-DA-KMnO_4_/LDPE were obtained and dried in a dehumidifier (DHF-PCL-01 plas-aid, made by Matsui) at 80 °C for 2 h.

Then, the LDPE films and LDPE containing 1, 3, and 5 wt% of particles were prepared by mixing the calculated amount of each masterbatch with adding neat LDPE via a single screw extruder (PolyLab™, HAAKE) with a blown film process. The extruder had a die gap of 1 mm and an outer diameter of 35 mm. The film thickness was controlled between 30 and 40 μm with a width of 12 cm. A standard metering screw (3:1) with 19 mm diameter and L/D of 25 was used in this process. The processing was conducted at 145 °C, 155 °C, 165 °C, and 165 °C for the fed, transfer, melt, and die zones, respectively. The screw speed was adjusted at 60 rpm and the pressure was controlled at 34.43 bar. The films containing 1, 3, and 5 wt% of particles were prepared by mixing the calculated amount of masterbatch with neat LDPE pallets and named with the code as follows: 1% DA/LDPE, 3% DA/LDPE, 5% DA/LDPE; 1% Alk-DA/LDPE, 3% Alk-DA/LDPE, 5% Alk-DA/LDPE; 1% KMnO_4_/LDPE, 3% KMnO_4_/LDPE, 5% KMnO_4_/LDPE; 1% DA-KMnO_4_/LDPE, 3% DA-KMnO_4_/LDPE, 5% DA-KMnO_4_/LDPE, 1% Alk-DA-KMnO_4_/LDPE, 3% Alk-DA-KMnO_4_/LDPE, and 5% Alk-DA-KMnO_4_/LDPE.

### 2.3 Characterization of films

#### 2.3.1 Attenuated Total reflectance-Fourier transform infrared (ATR-FTIR) analysis

Films containing DA, Alk-DA, KMnO_4_, DA-KMnO_4_, Alk-DA-KMnO_4_ were investigated for the interaction between LDPE and modified diatomite using Attenuated Total Reflectance-Fourier Transform Infrared (ATR-FTIR) spectroscopy (Thermo Scientific model Nicolet 6700). The film samples were placed on ATR crystal, and spectra were collected in the wavenumber range of 4000–500 cm^−1^ with a scan number of 32.

#### 2.3.2 Surface morphology analysis of films

The surface morphology of the films was examined using scanning electron microscopy (SEM, JCM-7000, NeoScopeTM Benchtop) at an accelerated voltage of 15 kV. Before the SEM analysis, the films were adhered to carbon double-sided tape and coated with a thin layer of gold‑palladium to avoid charging effects during exposure to the electron beam.

#### 2.3.3 Transparency property analysis

The transparency of films was analyzed using UV–Vis-NIR spectrophotometer (Carry UV–Vis-NIR, Agilent). The percentage of transmittance (%T) of the films was measured in the wavelength range of 400–800 nm. The experiments were repeated at least three times to obtain average values. The value of %T at the midpoint of visible range (600 nm) is used as the transparency value of the films.

#### 2.3.4 Thermal analysis of films

The thermal degradation of films was investigated using a Thermogravimetric Analyzer (Mettler Toledo, TGA/DSC 3+ STAR system). Approximately 5 mg of the sample was analyzed with a heating rate set at 10 °C/min, under nitrogen atmosphere at a flow rate of 20 mL/min.

The thermal properties of the films were analyzed by Differential scanning calorimetry (DSC) (METTLER Toledo, DSC 1 STAR System). All the samples were subjected to following method: first heating from 30 to 200 °C, cooling from 200 to 0 °C, and second heating from 0 to 200 °C with scanning rate of 10 °C /min under a constant nitrogen flow rate of 20 mL/min. The melting temperature (T_m_), crystallinity temperature (T_c_), melting enthalpy (∆H_m_), crystallinity enthalpy (∆H_c_), and percentage of crystallinity (% X_c_) were determined from DSC thermogram. % X_c_ was calculated using the following the eq. (1):

(1) $$\%X_{c}=\frac{∆H_{m}}{∆{H_{m}}^{o}}\times 100$$

#### 2.3.5 Mechanical properties analysis of films

The tensile strength and elongation at break of LDPE and films containing DA, Alk-DA, KMnO_4_, DA-KMnO_4_, and Alk-DA-KMnO_4_ were evaluated using a Universal Testing Machine (5ST Tinius Olsen) in accordance with the ASTM D882–18 standard. The tests were conducted with a preload of 1 N and grip separation speed of 300 mm/min. The samples were prepared in dumbbell shape following the ISO 37 (Type I). At least nine replicates were recorded for each condition to obtain average values.

#### 2.3.6 Ethylene removal properties analysis

The ethylene adsorption of LDPE, DA/LDPE, Alk-DA/LDPE, KMnO_4_/LDPE, DA-KMnO4/LDPE, and Alk-DA-KMnO_4_/LDPE was assessed using a Gas Chromatograph (Agilent 6890 N) equipped with a flame ionization detector (FID) and an HP-PLOT/Q column, which has an internal diameter of 0.53 mm and a length of 30 m. Approximately 0.4–0.5 g of each sample was placed in 20 mL glass jars, into which 600 μL of ethylene was injected. The jars were then incubated at 25 °C. At intervals of 0, 3, 12, 24, and 48 h, the headspace gas was collected from the jars and injected into the gas chromatograph. The resulting peaks were compared to a standard curve and their retention times. The amount of ethylene in the headspace was reported and calculated as μL/g following the eq. (2).

(2) $$\mathrm{Ethylene removal capacity}=\frac{\mathrm{initial volume}\;\left(\mu L\right)-\mathrm{remaining volume}\left(\mu L\right)\;}{\mathrm{Weight of films}\;\left(g\right)}$$

#### 2.3.7 Gas barrier properties of films

The oxygen transmission rate of LDPE, 1% DA/LDPE, and 1% Alk-DA-KMnO_4_/LDPE were determined using an Oxygen Transmission Rate Analyzer (OTR, OX-TRAN® Model 2/12) following the ASTM D-3985 standard at 23 °C, 0% RH.

The water vapor permeation rate of LDPE, 1% DA/LDPE, and 1% Alk-DA-KMnO_4_/LDPE were determined using a Water Vapor Permeation Analyzer (WVPR, MOCON, PERMATRAN-W® Model 3/34) following the ASTM F1249 standard at 38 °C, 90% RH.

### 2.4 Evaluation of the postharvest quality of bananas stored in LDPE bags

Banana fruits from Section 2.1.2 were divided into treatment groups: control (non-packed), neat LDPE, and optimized LDPE film formulations. In each treatment, eight fruits were individually packed in film bags (13 × 27 cm) to ensure uniform exposure to the packaging conditions. One non-packed control group and other packaging treatments were applied during storage. All samples were stored at 25 ± 2 °C and 60 ± 5% relative humidity for 10 days, and postharvest quality was evaluated on days 0, 1, 3, 6, 9, and 10.

#### 2.4.1 Peel color

Color was determined at three regions of the banana peel (near the stem end, middle, and blossom end) of each fruit using a colorimeter (Chroma Meter CR-400, Konica Minolta Sensing, Inc., Japan). The parameters measured were lightness (L), yellowness (b*), and hue angle. The average of the three measurements was recorded as the color value of each fruit.

#### 2.4.2 Weight loss

Banana fruits were weighed on day 0 and at each sampling date. Weight loss (%) of each fruit was calculated using Eq. (3):

(3) $$\mathrm{Weight loss}\;\left(\%\right)=\frac{W_{i}-W_{f}}{W_{i}}\times 100$$

Where $W_{i}$ is the initial weight of each fruit at day 0 (g), and $W_{f}$ is the weight at each storage date (g).

#### 2.4.3 Firmness

Fruit firmness was determined at three points on each fruit (near the blossom end, middle, and near the stem end) using a texture analyzer (TA-XT21Icon, England) with a 6 mm cylindrical probe at a testing speed of 1 mm/s. Firmness values were expressed as Newtons (N), and the average of the three points from both sides of the fruit was taken as the firmness of each banana.

#### 2.4.4 Total soluble solid (TSS)

Total soluble solids were measured using a handheld refractometer (PAL—S, Atago, Japan). Approximately 5 g of banana pulp from each fruit was blended with 10 mL of distilled water and centrifuged at 4000 rpm for 5 min. A drop of the supernatant was placed on the refractometer prism, and readings were recorded as °Brix. The values were multiplied by the dilution factor (×3) to obtain the final TSS as shown in eq. (4) (Wantat et al., 2021).

(4) $$\mathrm{Total Soluble Solid}=\%\mathrm{Brix}\times 3\;\left(\mathrm{dilution factor}\right)$$

### 2.5 Statistical analysis

All experimental procedures were performed in duplicate. Data were statistically analyzed using IBM SPSS Statistics (version 28 for Windows) via a one-way analysis of variance (ANOVA) followed by Tukey's post-hoc test, with statistical significance set at p ≤ 0.05. Results are expressed as mean ± standard deviation (SD), with SD values displayed as error bars in the figures.

## 3 Results and discussion

### 3.1 Attenuated Total reflectance-Fourier transform infrared (ATR-FTIR) analysis

The specific chemical functional groups present in raw diatomite (DA), alkaline-activated diatomite (Alk-DA), KMnO₄-impregnated Alk-DA (Alk-DA-KMnO_4_), and LDPE films containing these fillers were investigated by ATR-FTIR as illustrated in Fig. 1(A-C). As shown in Fig. 1 (A), the spectra of DA, Alk-DA, and Alk-DA-KMnO_4_ displayed characteristic Si-O-Si stretching (1093 cm^−1^), confirming the silica framework of diatomite. A smaller band at 617 cm^−1^, attributed to Si-O-Al stretching (Madejova´ et al., 2002), indicated the presence of alumina (Al_2_O_3_) in the diatomite structure (∼5%). (Ozdemir et al., 2022). In addition, the bands around 473 cm^−1^, 795 cm^−1^ and 1617 cm^−1^ correspond to the Si-OH translational vibration and the bending vibration of silanol group in phyllosilicate minerals (which are a type of sheet-like silicate mineral found in diatomite), as well as to the water in opal-A, respectively (Ahmadi et al., 2018; Ilia et al., 2009). The adsorption bands at 902 cm^−1^ and 925 cm^−1^ observed in the FTIR spectra of neat KMnO₄ are attributed to Mn—O stretching vibration in tetrahedral (Moon et al., 2015). Moreover, these spectra also appeared on the DA- KMnO_4_ and Alk-DA- KMnO_4_ without any shift in their position indicating no chemical interaction between diatomite and KMnO_4_. These observations were consistent with the XRD and XRF results shown in Figure S 1 and Table S 1, respectively. The XRD patterns of both DA-KMnO_4_ and Alk-DA-KMnO_4_ exhibited the characteristic diffraction peaks located at 2θ: 19.55, 23.93, 24.95, 25.88, 26.62, 27.57, 30.29,34.9079, 40.96${}^{{}^{\circ}}$, confirming the present of KMnO_4_ (Becerra et al., 2011; Chanka et al., 2024). Furthermore, the XRF analysis revealed a MnO content ranging from 18.52 to 18.72%. Additionally, immersing 10 g of Alk-DA in 100 mL of 2 N KMnO_4_ solutions yielded an uptake capacity of 2.82 ± 0.057 g/100 g (as shown in Figure S 2). This value is comparable to commercial sachets, which typically exhibit KMnO_4_ uptake ranging from 2.70 ± 0.05 g/100 g to 5.97 ± 1.43 g/100 g (Wills & Warton, 2004).

**Fig. 1:**
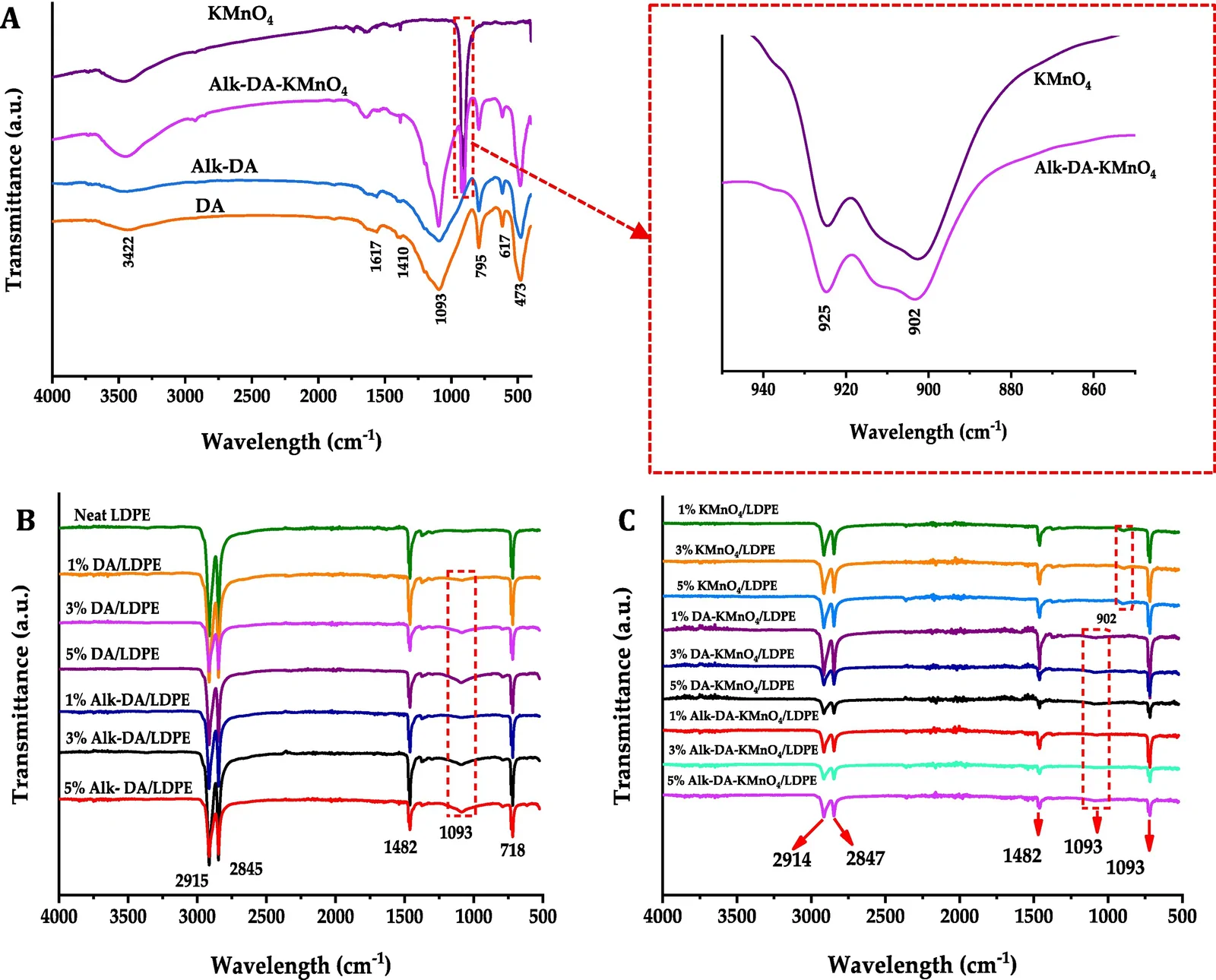
ATR-FTIR spectra of (a) DA, KMnO_4_, Alk-DA, Alk-DA-KMnO_4_, (b) Neat LDPE films, and LDPE films containing DA, and Alk-DA at 1, 3, and 5 wt%, and (c) LDPE films containing KMnO_4_, DA-KMnO_4_, and Alk-DA-KMnO_4_ at 1, 3, and 5 wt%.

The FTIR spectra of neat LDPE and LDPE films containing DA, Alk-DA, DA-KMnO_4_, and Alk-DA-KMnO_4_ at different loadings (Fig. 1 (B—C)) exhibited typical polymer bands at 2915, 2845 cm^−1^ (–CH₂– stretching), 1482 cm^−1^ (C—H bending in amorphous and crystalline phases), and 718 cm^−1^ (–CH₃ rocking), confirming that the LDPE matrix remained chemically intact (Basiron et al., 2019; Yaras et al., 2021). Incorporation of diatomite resulted in weak Si-O-Si bands at 1093 cm^−1^(Ilia et al., 2009; Madejova´ et al., 2002), while films containing KMnO₄ showed additional peaks at 902 cm^−1^, indicating the presence of permanganate (${{\mathrm{MnO}}_{4}}^{-})$ species (Becerra et al., 2011; Moon et al., 2015; Stella et al., 2014). Together, these results confirm the successful incorporation of diatomite and KMnO₄ into LDPE films without altering the polymer structure, with KMnO₄ retained in an active form as an ethylene scavenger.

### 3.2 Surface morphology analysis of films

The SEM micrographs of neat LDPE film and LDPE films containing 1 wt%, 3 wt%, and 5 wt% DA and Alk-DA were shown in Fig. 2. SEM images revealed that films containing 1 wt% DA and 1 wt% Alk-DA have a more uniform particle distribution compared to those with 3 wt% and 5 wt% loading. The surfaces of the LDPE films with 3 wt% and 5 wt% DA and Alk-DA displayed an increase in large agglomerates as the particle content rose. This is likely due to the incompatibility and weak interaction between the polar DA or Alk-DA and the non-polar LDPE matrix (Bikiaris et al., 2005; Liu & Kontopoulou, 2006). Furthermore, the irregular size and shape of the particles, as depicted in the SEM images, may have influenced their dispersion within the matrix, leading to the formation of clusters or agglomerates on the film surface. This observation aligns with findings reported by Joung et al. (2021). The cluster particles formed in DA were larger than those in Alk-DA, making them more difficult to disperse in the LDPE matrix during the extrusion process. Fig. 3 depicts the surface morphology of LDPE films with KMnO_4_ and impregnated particles (DA-KMnO_4_ and Alk-DA-KMnO_4_) at 1 wt%, 3 wt%, and 5 wt%. The SEM images indicate that larger aggregates are found in films with higher amounts of DA-KMnO_4_, Alk-DA-KMnO_4_, and KMnO_4_, especially at the 5 wt% content. Notably, KMnO_4_/LDPE showed more pronounced aggregation compared to DA-KMnO_4_/LDPE and Alk-DA-KMnO_4_/LDPE. Moreover, the presence of KMnO_4_ promotes particle aggregation, as observed in the comparison between Fig. 2 and Fig. 3. This increased aggregation is attributed to the mismatch between the highly polar KMnO_4_ and the non-polar LDPE matrix, which is consistent with the general incompatibility of polar fillers in non-polar matrix (Boonsiriwit et al., 2020; Joung et al., 2021).

**Fig. 2:**
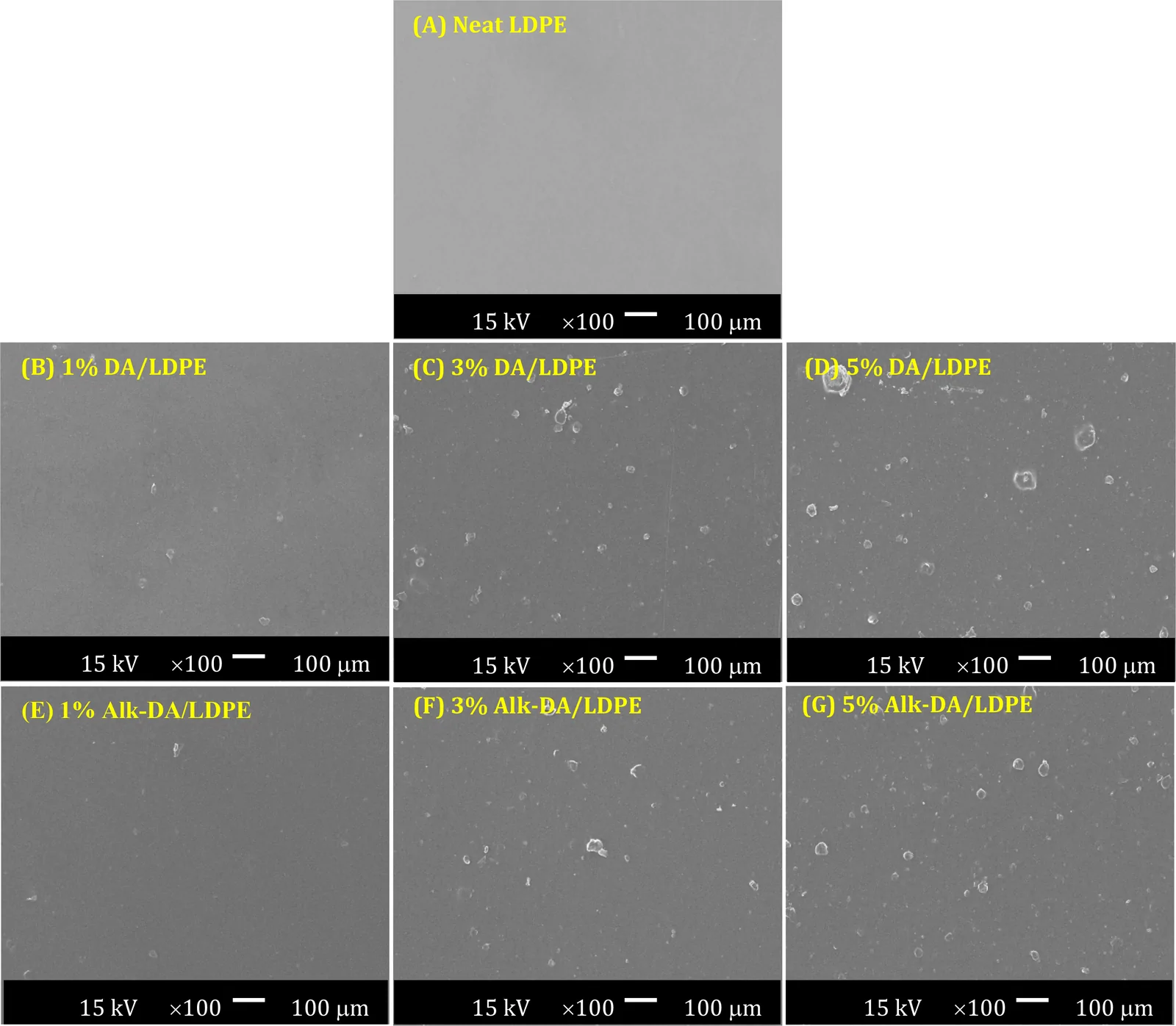
SEM micrographs of (A) Neat LDPE, (B) 1% DA/LDPE, (C) 3% DA/LDPE, (D) 5% DA/LDPE, (E) 1% Alk-DA/LDPE, (F) 3% Alk-DA/LDPE, and (G) 5% Alk-DA/LDPE films.

**Fig. 3:**
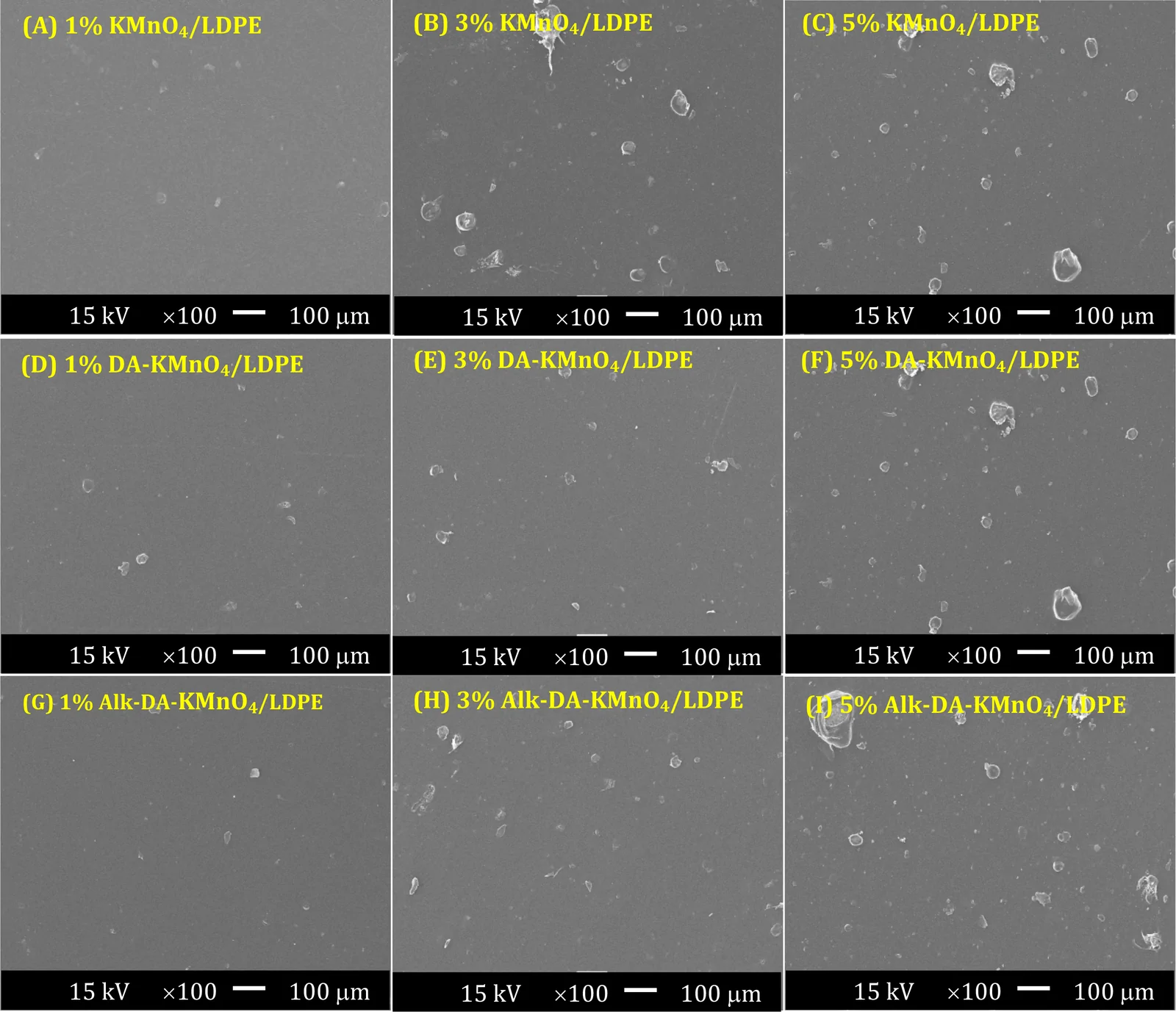
SEM micrographs of (A) 1% KMnO_4_/LDPE, (B) 3% KMnO_4_/LDPE, (C) 5% KMnO_4_/LDPE, (D) 1% DA- KMnO_4_/LDPE, (E) 3% DA- KMnO_4_/LDPE, (F) 5% DA- KMnO_4_/LDPE, (G) 1% Alk-DA- KMnO_4_/LDPE, (H) 3% Alk-DA- KMnO_4_/LDPE, (I) 5% Alk-DA- KMnO_4_/LDPE films.

### 3.3 Transparency property analysis

Transparency plays a crucial role in food packaging materials, commonly evaluated through transmittance and opacity measurements (Guzman-Puyol et al., 2022). In this research, the film transparency was examined using a UV–Vis spectrometer within the 400–800 nm wavelength range, with the percent transmittance at 600 nm serving as the transparency indicator (Balasubramanian et al., 2019; Du et al., 2021). Figure S 3 demonstrates the transparency of neat LDPE films and those containing 1 wt%, 3 wt%, and 5 wt% weight of DA, Alk-DA, KMnO_4_, DA-KMnO_4_, and Alk-DA-KMnO_4_. The results show that the particle content increases from 1 wt% to 5 wt%, the transparency of all particle-containing films decreases. However, the statistical analysis showed no significant difference between films containing 1 wt% of all those particles compared to the LDPE film (*p* > 0.05). In contrast, there were significant differences observed in films containing 3 wt% and 5 wt% of those particles (*p* < 0.05). This decline is likely attributed to the increased light scattering and absorption by the particles. Furthermore, the presence of agglomerates or clusters, as observed in SEM images, can intensify light scattering and reduce transparency. Nevertheless, the transparency of films like 1% DA/LDPE, 1% Alk-DA/LDPE, 1% DA-KMnO_4_/LDPE, and 1% Alk-DA-KMnO_4_/LDPE remains similar to neat LDPE, while 1% KMnO_4_/LDPE exhibits approximately 5% lower transparency than neat LDPE. These finding highlights that incorporating to 1 wt% DA and 1 wt% Alk-DA can maintain optical clarity, which is favorable for transparent food packaging applications.

### 3.4 Thermal properties of films

#### 3.4.1 Thermogravimetric analysis (TGA)

The thermal decomposition of neat LDPE films and LDPE films containing DA and Alk-DA at concentrations of 1 wt%, 3 wt%, and 5 wt% was analyzed using TGA to assess weight loss and derivative weight. The TGA and derivative curves for neat LDPE films and those containing DA and Alk-DA at 1 wt%, 3 wt%, and 5 wt% *w*/w are presented in Fig. 4 (A) and Figure S 4. The results indicate that both neat LDPE films and those containing DA and Alk-DA undergo a single-stage thermal degradation process. It occurred between 450 and 497 °C. The degradation of LDPE is attributed to the removal of hydrogen from the polymer chains. It results the formation of free radicals and a subsequent decrease in molecular weight that release the volatile products (García et al., 2009; Videira-Quintela et al., 2022). The presence of diatomite in all samples led to an increase in the onset temperature of LDPE, from 447 °C of neat LDPE increased to 459 °C as increased the amounts of DA and Alk-DA. This effect is likely due to the insulating properties of diatomite, which can slow down the degradation rate (Gokavi et al., 2020). However, the onset temperature was similar between DA/LDPE and Alk-DA/LDPE composites. Additionally, the degradation temperature (T_d_) and end-set temperature (T_end_) of LDPE showed minimal differences compared to LDPE containing DA or Alk-DA.

**Fig. 4:**
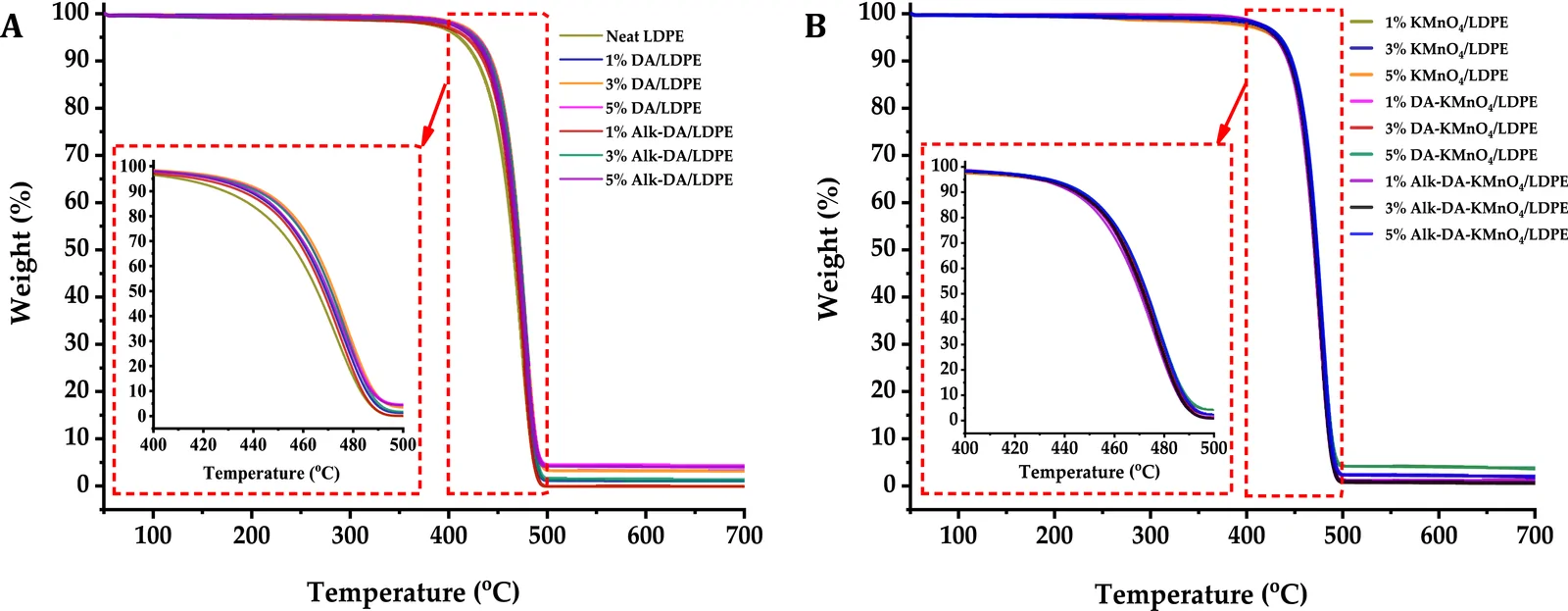
TGA curve of (A) neat LDPE films, LDPE films containing DA, and Alk-DA at 1, 3, and 5%, and (B) LDPE films containing KMnO_4_, and DA-KMnO_4_, Alk-DA-KMnO_4_ at 1, 3, and 5 wt%.

TGA curve and Derivative curve of LDPE films containing KMnO_4_, DA-KMnO_4_, and Alk-DA-KMnO_4_ at 1, 3, and 5 wt% are shown in Fig. 4 (B), and Figure S 5, respectively. The results show that films containing KMnO_4_, DA-KMnO_4_, and Alk-DA-KMnO_4_ had slightly higher onset and degradation temperatures around 2 to 5 °C compared to LDPE films with DA and Alk-DA. However, increasing the loading amount of DA-KMnO_4_ and Alk-DA-KMnO_4_ did not significantly affect the degradation temperature compared to LDPE containing 1 wt% of DA-KMnO_4_, and Alk-DA-KMnO_4_. These results indicate that even though diatomite consists good insulation properties, increasing loading amount of diatomite results to low dispersion of particle. This can create regions of poor interfacial adhesion and inefficient stress transfer, rendering it unsuitable to improve the degradation point of films (Kim et al., 2014). The lack of significant improvement in thermal stability with higher loadings may be attributed to the formation of agglomerates, which reduce the effective surface area of diatomite particles the diatomite particles interacting with the polymer matrix. Moreover, at the end degradation temperature of all those films was similar to that of LDPE films, at around 497 °C. In addition, diatomite is a non-flammable inorganic material within the temperature range where LDPE breaks down into volatile compounds. Therefore, the residue after completed decomposition of all films at 700 °C was the presence of diatomite.

#### 3.4.2 Differential scanning calorimetry (DSC) analysis

Differential Scanning Calorimetry (DSC) was used to evaluate the effect of the particle content on the melting temperature (T_m_), crystallization temperature (T_c_), fusion enthalpy (∆H_m_), and crystallinity (%X_c_) of neat LDPE films and LDPE films containing DA, Alk-DA, KMnO_4_, DA-KMnO_4_, and Alk-DA-KMnO_4_ at 1, 3, and 5 wt%. The thermogram from the first and the second heating cycle and as well as cooling cycle of LDPE films containing DA, And Alk-DA; and KMnO_4_, DA-KMnO_4_, and Alk-DA-KMnO_4_ at 1, 3, and 5 wt% are shown in the Fig. 5 (A-B), and Figure S 6, Figure S 7 and summarized data in Table S 2. The results show no shift in the endothermic or exothermic peak during the first and the second heating cycles, indicating no effect on the T_m_. However, there was an effect on the fusion enthalpy (∆H_m_) which was lower in the second heating. This could be attributed to improved organization, enhanced structure, and/or thicker crystals formed from stress-induced crystallization during the extrusion blown film process. This required a high fusion enthalpy to mobilize the polymer chain in the crystalline region during the first heating cycle (Sikora et al., 2019).

**Fig. 5:**
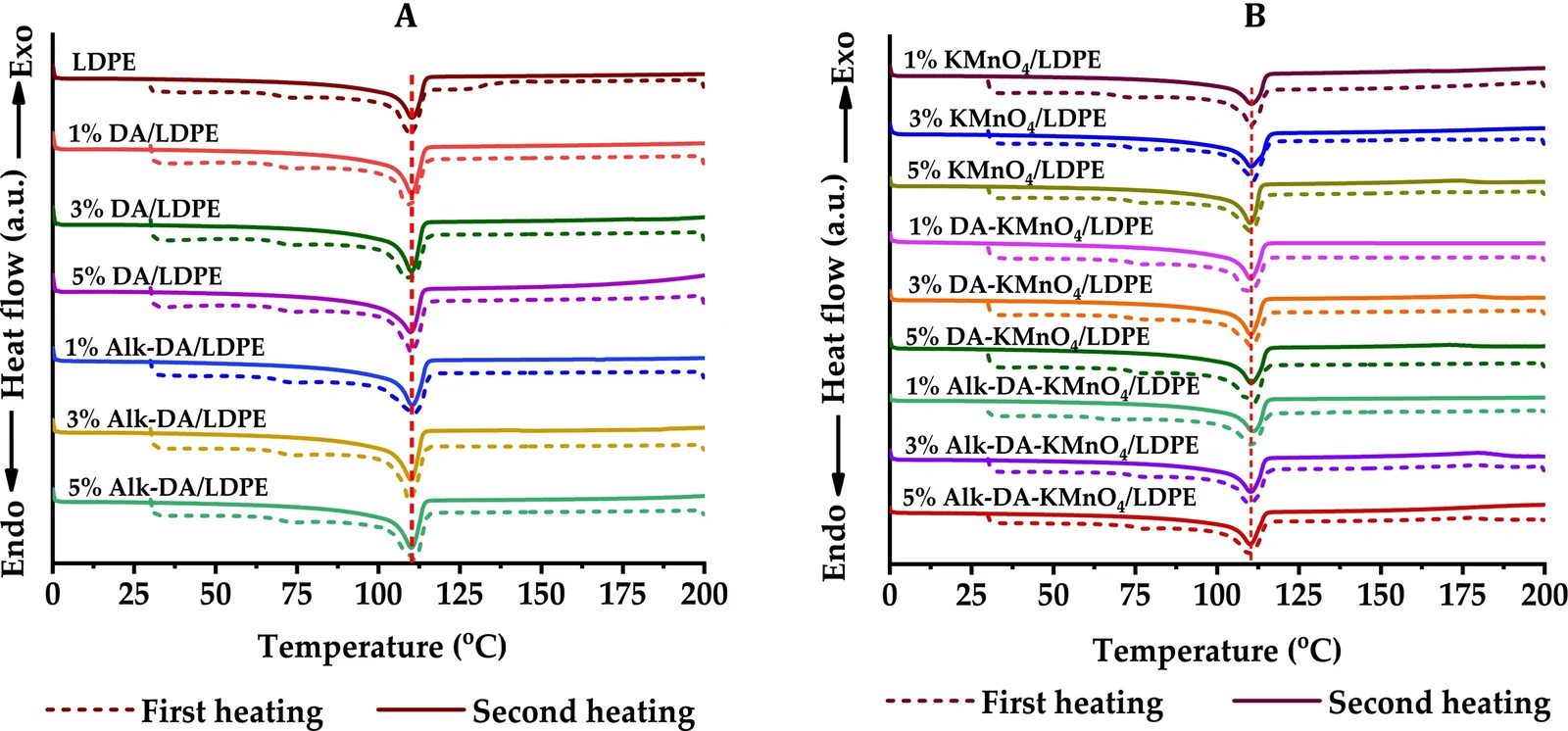
DSC thermogram of first and second heating cycle of (A) neat LDPE films, LDPE films containing DA, and Alk-DA at 1, 3, and 5%, and (B) LDPE films containing KMnO_4_, and DA-KMnO_4_, Alk-DA-KMnO_4_ at 1, 3, and 5 wt%.

Moreover, the enthalpy of melting (∆H_m_) of LDPE films containing DA and Alk-DA increased significantly with higher amount of these additives. Similarly, the percentage of crystallinity (%X_c_) in the LDPE films also increased as the amounts of DA and Alk-DA increased. These results demonstrated that the addition of particles may act as the nucleating agent and provide the nucleating effect, enhancing the crystallization of LDPE (Boonsiriwit et al., 2020). However, the %X_c_ of LDPE films containing 5% of KMnO_4_ and Alk-DA-KMnO_4_ slightly decreased compared to those containing 3 wt% of these particles. Overloading at 5 wt% of KMnO_4_ and Alk-DA-KMnO_4_ reduced their dispersibility, increased agglomeration in the LDPE matrix (as shown in Fig. 3). This led to a decrease in specific surface area and loss of the nucleation efficiency, similar to the case of Halloysite nanotube composites with PE (Boonsiriwit et al., 2020; Joung et al., 2021; Tas et al., 2017). Additionally, the %X_c_ of LDPE containing KMnO_4_, DA-KMnO_4_, and Alk-DA-KMnO_4_ were not significantly different compared to LDPE films containing DA, and Alk-DA. It revealed that KMnO_4_ impregnation did not affect these properties. Furthermore, the exothermic peak of LDPE films was not significantly affected by loading the particles. The exothermic peaks of all the films were observed at approximately 99 °C and 61 °C. This indicated that the addition of particles did not disturb the crystallization temperature of LDPE.

### 3.5 Mechanical properties analysis of films

The effect of different amounts of ethylene scavenger particles on the mechanical properties such as tensile strength and elongation of LDPE films, were evaluated. Fig. 6 (A) and (B) illustrate the tensile strength and elongation of LDPE films containing DA, Alk-DA, KMnO_4_, DA-KMnO_4_, and Alk-DA-KMnO_4_ at concentrations of 1, 3, and 5 wt%. The results indicated that the tensile strength and elongation of neat LDPE films significantly decreased as the particle content increased (*p* < 0.05). This reduction is likely due to particle aggregation associated with higher particle content. Similar decreases in the tensile strength of LDPE films with increased content of other particles, such as clay, have been reported by Joung et al. (2021), Boonsiriwit et al. (2020), and Sahraeian and Esfandeh (2016). Furthermore, LDPE films that contained KMnO_4_ had the lowest tensile strength and stretch. In contrast, LDPE films with Alk-DA demonstrated the highest tensile strength and elongation among the films containing these particles. When comparing DA and Alk-DA, LDPE films containing Alk-DA had higher strength and elongation. It may be due to the good distribution and less agglomeration of Alk-DA in the films as observed in SEM (Fig. 2). Additionally, films containing particles impregnated with KMnO_4_ showed lower tensile strength and elongation compared to films containing DA and Alk-DA. The tensile strength of LDPE films decreased by approximately 23.37%, 8.74%, 36.01%, 37.19%, 28.94% while elongation also decreased by approximately 38.85%, 27.22%, 40.70%, 30.14%, and 37.68% when loaded with 1% DA, 1% Alk-DA, 1% KMnO_4_, 1% DA-KMnO_4_, and 1% Alk-DA-KMnO_4_, respectively. This indicates that the aggregated formation of KMnO_4_ and the impregnated particles (DA-KMnO_4_ and Alk-DA-KMnO_4_) significantly affect the tensile strength and elongation of LDPE films. Moreover, higher loading of KMnO_4_ and impregnated particles led to poor dispersion and the formation of large particle aggregations in the LDPE. However, the tensile strength of LDPE films with 1% Alk-DA-KMnO_4_, DA, and Alk-DA reached levels between 15 and 19 MPa, which is satisfactory and comparable to the commercial LDPE films available for fresh fruits and vegetables in the market (Boonsiriwit et al., 2020).

**Fig. 6:**
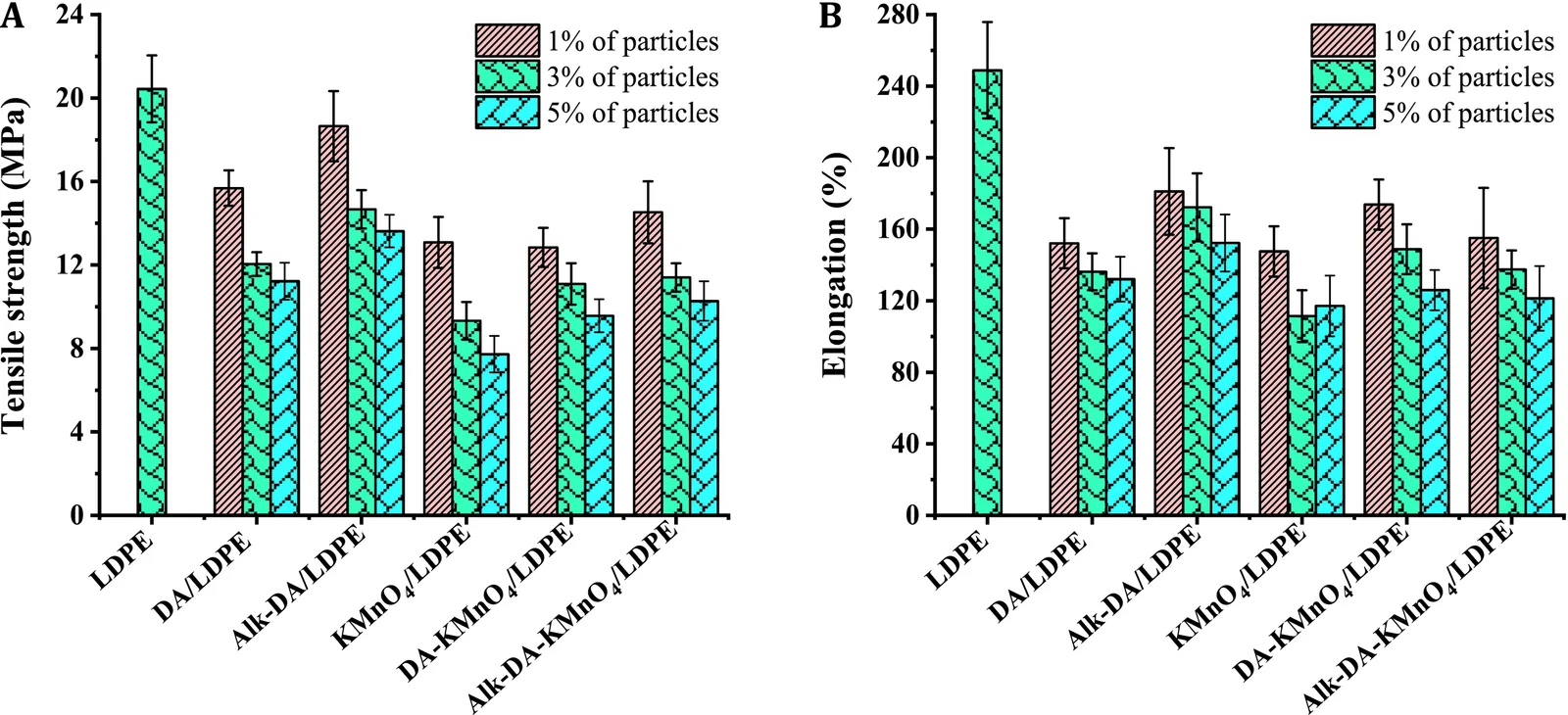
Tensile Strength (A), and Elongation (B) of Neat LDPE films and LDPE films containing DA, Alk-DA, KMnO_4_, DA-KMnO_4_, and Alk-DA-KMnO_4_ are 1, 3, and 5 wt%.

### 3.6 Ethylene removal properties analysis

In the powder form, the ethylene removal capability of DA, Alk-DA, KMnO_4_, and Alk-DA-KMnO_4_ at 3, 12, 24, and 48 h incubation was shown in Fig. 7 (A). The capacity of all samples to remove ethylene was significantly impacted by the initial concentration of ethylene after 3 h of incubation (*p* < 0.05). The Alk-DA-KMnO_4_ showed the highest performance of ethylene removal about 909.49 ± 29.98 μL/g after 24 h (*p* < 0.05). Furthermore, Alk-DA was more effective at removing ethylene than DA, likely due to NaOH increasing the surface area of diatomite. These results were consistent with the results of BET analysis as shown in Table S 3. The ethylene removal capability increased with increasing specific surface area (Erdoğan & Akbelen, 2012). Moreover, the ethylene removal capacity of KMnO_4_ powder was significantly lower than that of the impregnated particles. This may be associated with the limited gas-accessible surface of neat KMnO_4_ particles and their possible agglomeration. In case of Alk-DA-KMnO_4_, the porous Alk-DA carrier may increase the distribution and accessibility of KMnO_4_ while also providing sites for physical adsorption. Thus, the high removal observed for Alk-DA-KMnO₄ may be associated with the combined contributions of physical adsorption by Alk-DA and possible oxidation ethylene by KMnO₄ as also discussed in others' work (Awalgaonkar et al., 2020; Wills & Warton, 2004). However, oxidation products and ethylene mass balance were not determined. Therefore, oxidation is proposed only as possible mechanism.

**Fig. 7:**
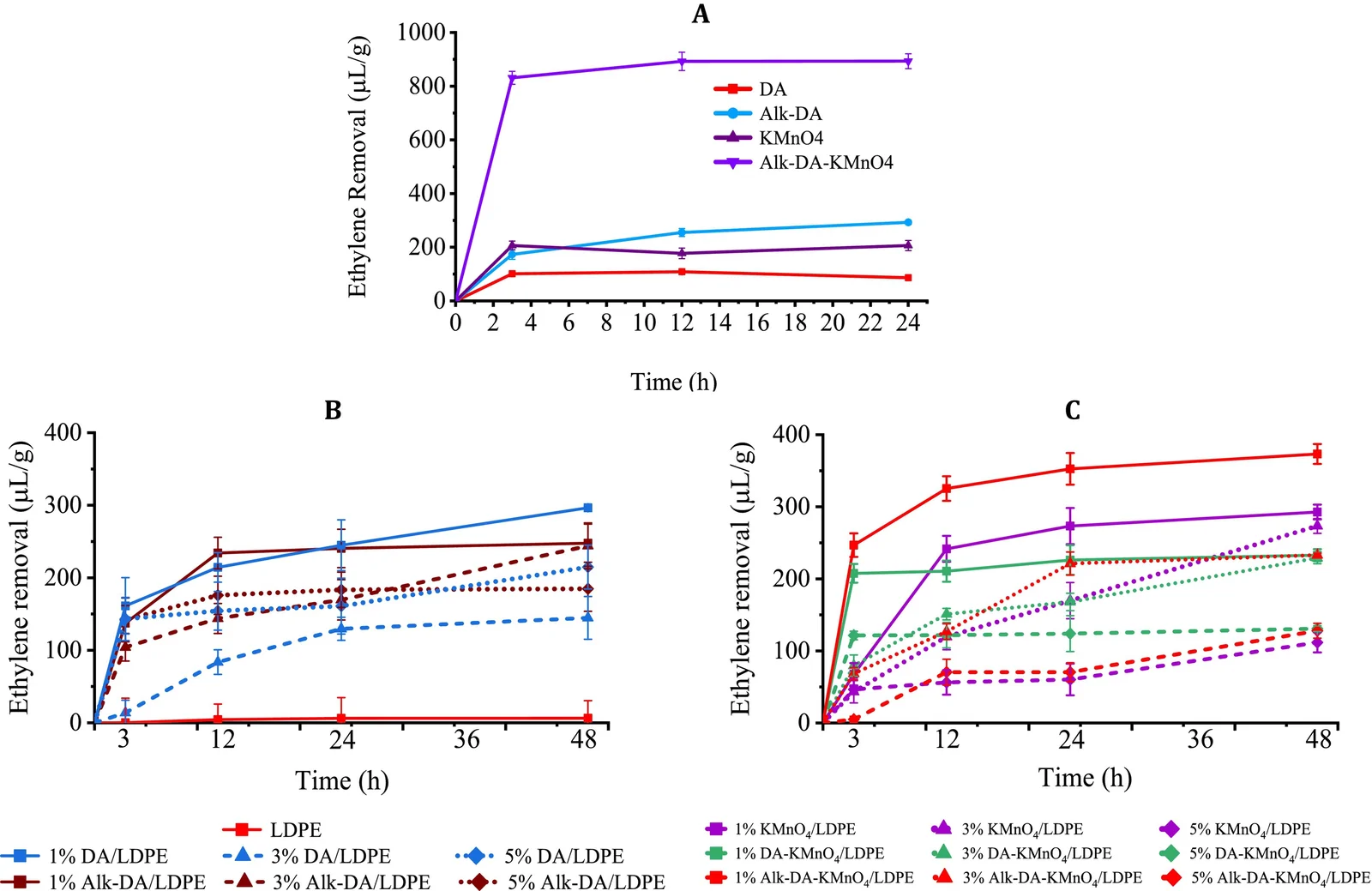
Ethylene removal of (A) DA, Alk-DA, KMnO_4_ and Alk-DA-KMnO_4_ particle, (B) Neat LDPE film, and LDPE films containing DA, and Alk-DA at 1, 3, and 5 wt%, and (C) LDPE films containing KMnO_4_, DA-KMnO_4_, and Alk-DA-KMnO_4_ at 1, 3, and 5 wt%.

After incorporation into LDPE, the ethylene removal of the neat LDPE films, and LDPE films containing DA, Alk-DA, KMnO_4_, DA-KMnO_4_ and Alk-DA-KMnO_4_ at 1, 3, and 5 wt% were evaluated by measuring the remaining amount of ethylene in the headspace of the test vial after 3, 12, 24, and 48 h of incubation as illustrated in the Fig. 7 (B—C). The films with ethylene scavenger particles demonstrated a significantly greater capacity for ethylene removal compared to neat LDPE films (*p* < 0.05). However, when the particle content was increased to 3 wt% and 5 wt%, the ethylene removal capacity of the films decreased compared to that of the film containing 1 wt% content (*P* < 0.05). The LDPE film with 1 wt% Alk-DA-KMnO_4_, and 1 wt% DA exhibited the highest ethylene removal capacity, reaching 373.22 ± 13.83 μL/g, and 296.58 ± 2.07 μL/g at 48 h, respectively. The effectiveness of ethylene removal in these films is influenced by the concentration and distribution of the ethylene scavenging agents (Ebrahimi et al., 2021; Joung et al., 2021). The decrease in ethylene removal capacity in films containing 3 wt% and 5 wt% loading is likely due to particle aggregation at high loading (Wang & Ajji, 2022b). These finding align with the SEM results presented in Fig. 2, and Fig. 3, which indicate that the uniform dispersion of particles at a 1 wt% concentration leads to better ethylene removal, while larger particle clusters at 3 wt% and 5 wt% concentrations reduce this capacity. Furthermore, the ethylene removal performance of LDPE films containing impregnated particles (DA-KMnO_4_, Alk-DA-KMnO_4_) was significantly lower than that of Alk-DA-KMnO_4_ powder. Additionally, incorporating the impregnated particles (DA-KMnO_4_, Alk-DA-KMnO_4_) into LDPE films led to a marked reduction in ethylene removal efficiency compared to the Alk-DA-KMnO_4_ powder (*p* < 0.05). The unconstrained powder benefits from a high surface area and accessible mesoporous channels, promoting unhindered mass transfer of ethylene to the KMnO_4_ reaction sites. In contrast, the melt-compounding process causes molten LDPE to coat the particles and penetrate their pores, blocking active sites (Rozaini et al., 2024). This issue is further compounded by poor interfacial compatibility between the polar mineral filler and the nonpolar polymer matrix, which induces particle aggregation and restricts gas transport to the interior of the clusters (Roy et al., 2011). The result indicated that 1% Alk-DA-KMnO_4_/LDPE, and 1% DA/LDPE were highest ethylene removal capacity which selected to evaluate the shelf-life of fresh fruits. The LDPE films containing 1 wt% DA and 1 wt% Alk-DA-KMnO_4_ demonstrated superior ethylene removal compared to other films examined in this study. Additionally, the appearance and mechanical properties of these LDPE films were deemed satisfactory. Consequently, it can be concluded that LDPE films with 1 wt% DA and 1 wt% Alk-DA-KMnO_4_ have the potential for use in packaging fresh fruits. These films were chosen for further investigation into their gas barrier properties and storage performance.

### 3.7 Gas barrier properties of films

The films presenting higher ethylene removal capacity were selected to evaluate the oxygen transmission rate (OTR) and water vapor permeation rate (WVPR) compared with LDPE. In Figure S 8, and Table S 4 show the OTR and WVPR of LDPE, 1% DA/LDPE, 1% Alk-DA-KMnO_4_/LDPE. The results indicated that the incorporation of DA and Alk-DA-KMnO_4_ particles did not enhance the gas barrier properties of the films as it led to increased oxygen transmission. There were significant differences between OTR of LDPE and 1% DA/LDPE (*p* < 0.05). The OTR values of 1% DA/LDPE, and 1% Alk-DA-KMnO_4_/LDPE increased by approximately 131.62% and 4.42%, respectively, compared to LDPE. meanwhile, the WVPR of 1% DA/LDPE, and 1% Alk-DA-KMnO_4_/LDPE slightly increased, by approximately; 16.92% and 8.41%, respectively (*p* > 0.05). This observed increase is possibly due to diatomite being inherently hydrophilic due to abundant surface silanol (Si-OH) groups (as shown in Fig. 1A), creating a thermodynamic mismatch with the non-polar, hydrophobic LDPE matrix. The poor interfacial adhesion between diatomite and LDPE matrix leads to debonding and forms micro voids. These interfacial voids cause accelerated molecular diffusion and convective gas transport. Moreover, the polar interaction of diatomite tends to promote localized agglomerate formation with the polymer melt. These aggregates disrupt the uniform packing of LDPE chain, leading to defects that compromise barrier integrity (Boonsiriwit et al., 2020; Joung et al., 2021; Wang & Ajji, 2023).

### 3.8 Evaluation of the postharvest quality of bananas stored in optimized LDPE bags

For the evaluation of postharvest quality, one non-packed group (control) and three packaging treatments were applied during storage. The treatments included LDPE bags made from neat LDPE, 1% DA/LDPE, and 1% Alk-DA-KMnO_4_/LDPE. In each treatment, eight fruits were individually packed to ensure uniform exposure to the packaging conditions.

#### 3.8.1 Peel color

According to Altaf et al. (2020), Santoyo-Mora et al. (2019), and Ringer et al. (2018) banana ripening is defined by a seven-stage progression of peel color changes, shifting from solid green to yellow with brown spots. The process begins at Stage 1 as an unripe, completely green banana, which transitions to light green in Stage 2 as ripening initiates. By Stage 3, the fruit remains underripe with more green tones than yellow, before reaching Stage 4, where a yellow peel with a green tip signals the start of ripeness. Stages 5 and 6 represent fully ripe fruit, characterized by bright yellow and pale-yellow hues, respectively. Finally, Stage 7 marks the overripe phase, identified by a yellow peel speckled with brown spots.

Peel color changes of bananas stored under different packaging conditions are shown in Fig. 8 (A). The measured parameters included lightness (L*), yellowness (b*), and hue angle is presented in Figure S 9 (A-C). The progression of peel color in bananas during storage was strongly influenced by the type of packaging. In the control (non-packed) and LDPE groups, rapid color changes were observed, with peel color shifting from green to yellow as storage progressed, showing yellowing by day 6, as indicated by increased L* and b* values and decreased hue angle. By day 9–10, these fruits exhibited advanced ripening, characterized by browning and dark spot formation. The control group exhibited a slight change to light green-yellow with dark spot appearing on the peel and at the blossom end, with the dried peel skin on day 6, accompanied by localized peel drying. This deterioration is likely related to the accumulation of reactive oxygen species (ROS) when bananas are exposed to air, triggering oxidative enzymes that cause browning and spot formation (Pongprasert et al., 2021).

**Fig. 8:**
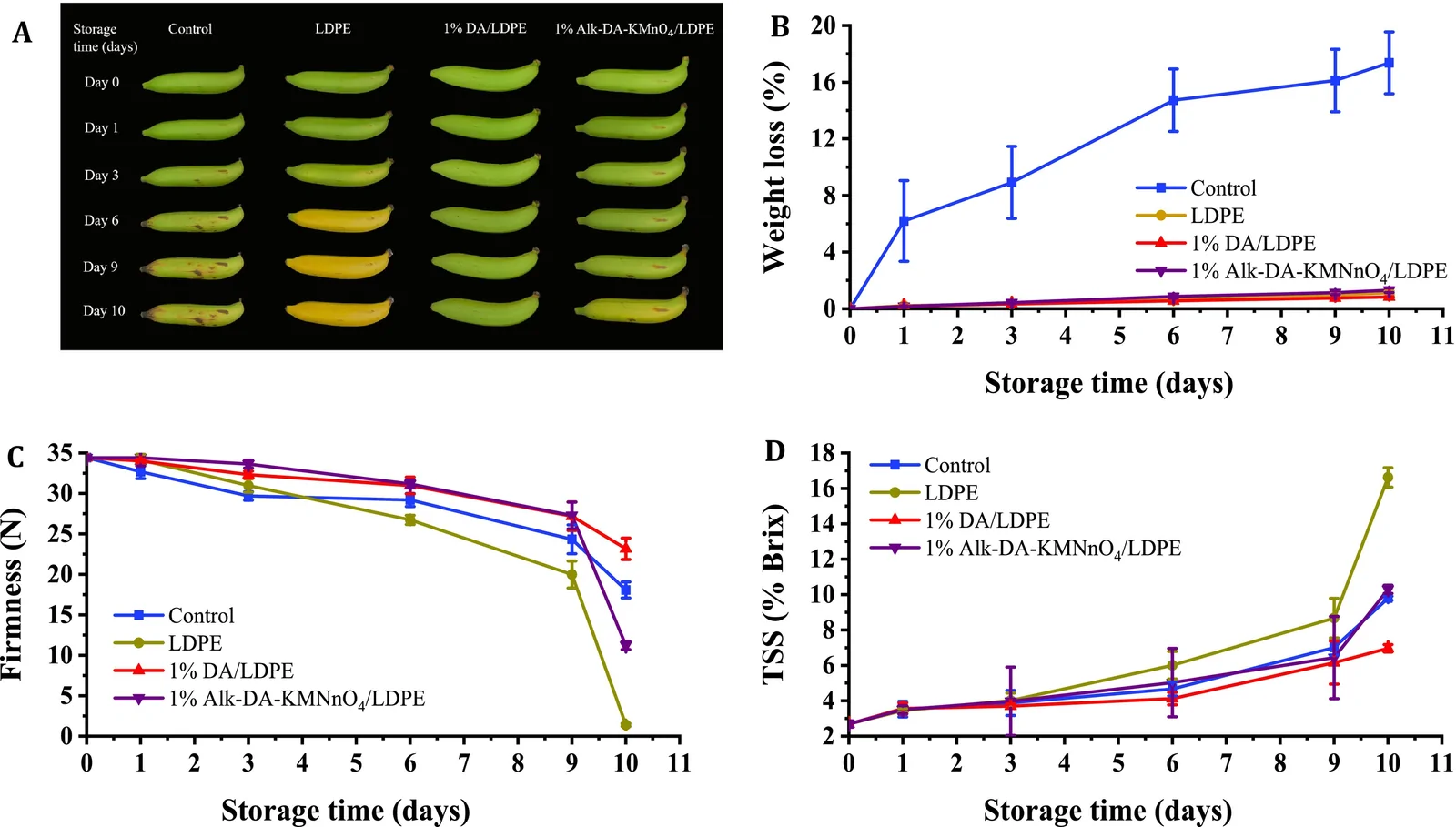
(A) the peel color change, (B) Weight loss, (C) Firmness, (D) TSS of bananas during storage at 25 ± 2 °C of control (non-packed), and packed in LDPE, 1% DA/LDPE, and 1% Alk-DA-KMnO_4_/LDPE films.

Bananas stored in 1% DA/LDPE maintained their green appearance until day 10, with only slight changes in color parameters. This indicates an effective reduction of ethylene action and delayed chlorophyll degradation (Tepamatr, 2023). Similar roles of suppressed ethylene activity and slowed chlorophyll breakdown in extending the shelf life of climacteric fruits have also been reported in previous studies(Ebrahimi et al., 2021; Joung et al., 2021; Wang et al., 2022). Fruits stored in 1% Alk-DA-KMnO_4_/LDPE showed an intermediate response, retaining a green-yellow color (stage 3) with moderate changes in peel color values.

Although the closed-vial experiment provides a controlled comparison among the film formulations, it did not reproduce the dynamic atmosphere in packages containing bananas. In an actual package, ethylene, O₂ and CO₂ concentrations change as a result of respiration and gas permeation. Therefore, the ethylene-removal results obtained from the closed-vial test should not be considered direct measurements of ethylene in packages. The observed delay in banana ripening may result from the combined effects of ethylene interaction with the films, gas permeability, moisture regulation, and fruit-to-fruit biological variability. Simultaneous monitoring of in-package ethylene, O₂, and CO₂ will be required to distinguish these contributions in future studies.

These findings indicate that 1% DA/LDPE was the most effective treatment in delaying peel color change, followed by 1% Alk-DA-KMnO_4_/LDPE, whereas neat LDPE showed minimal improvement compared to the control.

#### 3.8.2 Weight loss

The weight loss of bananas during storage is illustrated in Fig. 8 (B). Non-packed bananas (control) exhibited a rapid and significant increase in weight loss throughout the storage period, with clear differences compared to all packaged groups (*p* < 0.05). Significant differences in weight loss were observed on days 1, 3, and 6, while by days 9 and 10, the rate of weight loss in the control group stabilized but remained substantially higher than in the packaged treatments. By the end of the storage period, the control group experienced a weight loss of nearly 18% of their initial weight. In contrast, bananas packed in LDPE, 1% DA/LDPE, and 1% Alk-DA-KMnO_4_/LDPE exhibited markedly lower weight losses, remaining within 2–3% of their initial weight by day 10. Among these, 1% DA/LDPE showed the lowest cumulative weight loss, followed by LDPE and 1% Alk-DA-KMnO_4_/LDPE. Although differences among the packaged treatments were not statistically significant, the slightly better performance of 1% DA/LDPE suggests an enhanced ability to reduce moisture loss through the peel, likely due to reduced transpiration and evaporation rates (Boonsiriwit et al., 2020). The substantial weight loss observed in non-packed bananas is likely due to higher water loss through transpiration and respiration, leading to reduced freshness, shriveling, and the development of dry, wrinkled peel, as observed in Fig. 8 (A). This aligns with previous studies indicating that increased water loss during storage diminishes fruit quality and consumer acceptability (Nur Faidah Rahman et al., 2021).

#### 3.8.3 Firmness

Changes in banana firmness during storage are presented in Fig. 8 (C). Firmness decreased in all treatments over time, reflecting the natural softening process during ripening. Statistical analysis showed no significant differences among treatments on days 1, 3, and 6. From day 6 onward, bananas packed in LDPE softened more rapidly, showing significantly lower firmness than the other treatments by days 9 and 10 (*p* < 0.05). Bananas stored in 1% Alk-DA-KMnO_4_/LDPE maintained firmness up to day 9 but exhibited a notable decline on day 10, whereas those in 1% DA/LDPE showed only a slight reduction in firmness throughout the storage period. The accelerated softening in LDPE treatments may be attributed to ethylene accumulation, which enhances pectin degradation in the cell wall and accelerates starch hydrolysis into soluble sugars during ripening (Joung et al., 2021; Nur Faidah Rahman et al., 2021; Salvador et al., 2007). Therefore, the ability of 1% DA/LDPE to maintain both the appearance and firmness of bananas reveal its potential as an effective packaging material.

#### 3.8.4 Total soluble solid (TSS)

TSS is a crucial quality parameter for fruits, primarily composed of sugars with small amounts of proteins, amino acids, and organic substances, serving as an indicator for fruit sweetness (Kusumiyati et al., 2020). TSS increased progressively in all treatments during storage (Fig. 8 (D)). Statistical analysis revealed no significant differences in TSS among test groups on days 1 and 3 (*p* > 0.05). In contrast, TSS of bananas packed in LDPE films was significantly higher than that of other test groups on days 6, 9, and 10 (*p* < 0.05). Notably, the TSS of bananas packed in 1% DA/LDPE remained the lowest among all test groups throughout the storage period. The initial TSS of all test banana groups was 2.69% Brix. On day 10, TSS increased to 9.80, 16.63, 6.97, and 10.30% Brix for non-packed bananas (control), bananas packed in LDPE, 1% DA/LDPE and 1% Alk-DA-KMnO_4_/LDPE, respectively. These results suggest that bananas packaged in 1% DA/LDPE showed a slower increase in TSS during storage. This observation may indicate slower changes in soluble solids associated with the ripening process (Behera et al., 2025; Joung et al., 2021).

Although 1% Alk-DA-KMnO_4_/LDPE exhibited higher ethylene removal, the overall postharvest evaluation demonstrated that 1% DA/LDPE was more effective in extending banana shelf-life up to 10 days. This result can be attributed to the balance between ethylene removal and gas barrier properties (OTR, and WVPR) and fruit respiratory physiology. The higher OTR in the 1% DA/LDPE film allowed greater O_2_ transfer through the film, potentially helping to maintain aerobic respiration. In contrast, high O_2_ barrier value under some packaging conditions, may promote physiological stress, metabolic off-flavors, and peel color change (Cui et al., 2025). However, this proposed explanation could not be confirmed in the present study because the concentration of O_2_ and indicators of anaerobic metabolism were not measured. Moreover, the higher WVPR of 1% DA/LDPE film facilitated the controlled release of transpired water vapor, preventing saturated relative humidity and liquid condensation on the fruit surface. In contrast, films with lower WVPR risk moisture accumulation at the boundary layer, promoting peel softening, lenticel breakdown (Cui et al., 2025; Kader et al., 1989).

## 4 Conclusions

These results partially supported the proposed hypotheses. Alkaline activation improved the ethylene removal of diatomite (Alk-DA). Alk-DA-KMnO_4_ exhibited the highest removal in both powder and LDPE-based forms. After incorporation into LDPE, the performance of those particles decreased. This is because the accessibility of ethylene is limited. The measured ethylene reduction may involve physical adsorption by diatomite and possible KMnO_4_ oxidation. However, oxidation was not directly confirmed. The 1% DA/LDPE and 1% Alk-DA-KMnO_4_/LDPE films achieved ethylene removal efficiencies of 296.58 ± 2.07 μL/g, and 373.22 ± 13.83 μL/g, respectively. Thereby prolonging the postharvest ripening of packaged bananas compared to neat LDPE film. However, higher particle loadings compromised mechanical properties of films due to particle agglomeration and did not enhance gas barrier properties, despite slight improvements in thermal stability and crystallinity. These composite films showed proof of concept potential for postharvest ethylene removal. Although Alk-DA-KMnO₄/LDPE showed the highest ethylene removal, 1% DA/LDPE most effectively maintained the measured banana quality attributes during the 10-day storage period. These findings indicate that better postharvest performance was not governed solely by ethylene removal but also influenced by overall properties of the films. However, their practical and commercial applicability remains unverified. Long-term matrix stability, migration of KMnO_4_ or Mn from films, and overall food-contact safety were not evaluated. Therefore, these films should be regarded as laboratory scale and cannot yet be considered suitable for direct food contact or commercial fresh-fruit packaging.

## Author contribution

Theara Yann conducted the whole experiment, analysis, and wrote the manuscript. Orathai Boondamnoen conceived the study, design and supervised the experiments, revised the manuscript. Panita Ngamchuachit advised the experiments, provided the lab to conduct experiment, revised the manuscript. Phanny Yos and Charinee Winotapun advised the study and experiments. Lee Hwei Voon, and Yew Hoong Wong advised during conducting overseas lab research at Universiti Malaya.

## CRediT authorship contribution statement

**Theara Yann:** Writing – original draft, Methodology, Formal analysis, Data curation. **Charinee Winotapun:** Conceptualization. **Phanny Yos:** Conceptualization. **Lee Hwei Voon:** Conceptualization. **Yew Hoong Wong:** Conceptualization. **Panita Ngamchuachit:** Investigation, Conceptualization. **Orathai Boondamnoen:** Writing – review & editing, Supervision, Investigation, Funding acquisition.

## Funding

This research was supported by the Thailand Science Research and Innovation Fund Chulalongkorn University (BCG_FF_68_255_2300_062), and the Second Century Fund (C2F), Chulalongkorn University. The Department of Materials Science, Research grant support of International Research Partnership (IRP2024) by Faculty of Science, Chulalongkorn University in Thailand, and the Higher Education Institution Project, Institute Technology of Cambodia (ITC).

## Declaration of competing interest

The authors declare that they have no known competing financial interests or personal relationships that could have appeared to influence the work reported in this paper.

## Data Availability

Data will be made available on request.
