# Supplementary material for: Effect of diatomite and KMnO4-impregnated diatomite in LDPE films on ethylene removal and postharvest quality of bananas
Source: Food Chem X. 2026 Sep 26;39:104499. doi: 10.1016/j.fochx.2026.104499 (PMC13639747; doi:10.1016/j.fochx.2026.104499)
Supplement: Supplementary file 1 [file mmc1.docx]

**Supplementary materials**

**Figure S 1.** XRD patterns of Raw diatomite (DA), DA-KMnO_4_, and Alk-DA- KMnO_4_

**Figure S 2.** KMnO_4_ uptake of Alk-DA at different concentration of KMnO_4_ within different amount of Alk-DA

**Figure S 3.** Transparency of LDPE films and LDPE films containing DA, Alk-DA, KMnO_4_, DA-KMnO_4_, and Alk-DA-KMnO_4_ at 1, 3, and 5 wt%.

**Figure S 4.** Derivative curve of neat LDPE films, LDPE films containing DA, and Alk-DA at 1, 3, and 5 wt%

**Figure S 5.** Derivative curve of LDPE films containing KMnO_4_, and DA-KMnO_4_, Alk-DA-KMnO_4_ at 1, 3, and 5 wt%.

**Figure S 6.** DSC thermogram of cooling cycle of neat LDPE films, LDPE films containing DA, and Alk-DA at 1, 3, and 5 wt%.

**Figure S 7.** DSC thermogram of cooling cycle of LDPE films containing KMnO4, and DA-KMnO_4_, Alk-DA-KMnO_4_ at 1, 3, and 5 wt%.

**Figure S 8.** OTR and WVPR of LDPE, 1% DA/LDPE, and 1% Alk-DA-KMnO_4_/LDPE

**Figure S 9.** (A) The lightness, (B) Yellowness, and (C) Hue angle of bananas during storage at 25 ± 2 ^o^C of control (non-packed), and packed in LDPE, 1% DA/LDPE, and 1% Alk-DA-KMnO_4_/LDPE films.

**A**

**B**

**C**

**Table S 1.** The chemical composition of Raw diatomite (DA), DA-KMnO_4_, and Alk-DA- KMnO_4_

| Samples | SiO_2_ | Al_2_O_3_ | MnO | P_2_O_5_ | K_2_O | CaO | Fe_2_O_3_ |
| --- | --- | --- | --- | --- | --- | --- | --- |
| DA | 91.15 | 1.86 | 0.05 | 1.17 | 0.79 | 1.06 | 3.17 |
| DA-KMnO_4_ | 66.23 | 1.61 | 18.52 | 0.82 | 0.49 | 9.98 | 0.60 |
| Alk-DA- KMnO_4_ | 66.09 | 1.45 | 18.72 | 0.81 | 0.46 | 9.90 | 0.57 |

**Table S 2** Thermal characteristics of films.

| **Sample** | **Thermal analysis** | | | **First heating** | | **Second heating** | | | |
| --- | --- | --- | --- | --- | --- | --- | --- | --- | --- |
|  | **Onset T_o_**  **(^o^C)** | **Degradation T_d_**  **(^o^C)** | **End set T_end_ (^o^C)** | **Melting T_m_**  **(^o^C)** | **Fusion enthalpy ∆H_m_ (j/g)** | **Melting T_m_**  **(^o^C)** | **Fusion enthalpy ∆H_m_ (j/g)** | **Crystallinity Xc**  **(%)** |  |
| LDPE | 447.73 ± 0.77^a^ | 476.20 ± 1.79^a^ | 496.71 ± 1.37^a^ | 109.31 ± 0.22^ab^ | 143.86 ± 0.43^f^ | 110.18 ± 0.13^a^ | 97.53 ± 0.25^a^ | 33.29 ± 14^a^ |  |
| 1% DA/LDPE | 452.12 ± 0.25^b^ | 478.40 ± 1.22^a^ | 497.23 ± 0.11^a^ | 109.50 ± 0.35^a^ | 110.56 ± 0.78^b^ | 109.99 ± 0.70^a^ | 107.69 ± 0.54^c^ | 36.75 ± 0.25^bc^ |  |
| 3% DA/LDPE | 456.03 ± 0.40^b^ | 479.59 ± 1.24^a^ | 496.44 ± 1.38^a^ | 109.67 ± 0.47^ab^ | 119.55 ± 2.54^d^ | 109.81 ± 0.57^a^ | 111.14 ± 1.67^cd^ | 37.93 ± 0.80^c^ |  |
| 5% DA/LDPE | 457.75 ± 0.91^d^ | 478.79 ± 1.50^a^ | 497.1 ± 0.05^a^ | 109.65 ± 0.46^ab^ | 126.35 ± 4.47^e^ | 109.68 ± 0.48^a^ | 120.08 ± 3.00^de^ | 40.98 ± 1.45^d^ |  |
| 1% Alk-DA/LDPE | 452.99 ± 0.36^b^ | 477.44 ± 0.66^a^ | 495.61 ± 0.13^a^ | 110.82 ± 0.58^b^ | 114.91 ± 0.81^cd^ | 110.13 ± 0.09^a^ | 114.84 ± 0.57^d^ | 39.19 ± 0.27^cd^ |  |
| 3% Alk-DA/LDPE | 457.63 ± 0.77^d^ | 479.71 ± 0.98^a^ | 497.13 ± 0.13^a^ | 109.29 ± 0.21^ab^ | 116.97 ± 2.48^cd^ | 109.80 ± 0.57^a^ | 118.77 ± 1.78^de^ | 40.54 ± 0.86^d^ |  |
| 5% Alk-DA/LDPE | 459.32 ± 0.29^e^ | 477.22 ± 0.74^a^ | 496.77 ± 0.85^a^ | 110.61 ± 0.43^b^ | 122.59 ± 4.33^de^ | 109.77 ± 0.54^a^ | 121.64 ± 3.04^e^ | 41.52 ± 1.47^d^ |  |
| 1% KMnO_4_/LDPE | 457.26 ± 0.84^d^ | 479.82 ± 1.17^a^ | 496.63 ± 0.35^a^ | 109.98 ± 0.69^ab^ | 111.93 ± 0.79^bc^ | 110.34 ± 0.24^a^ | 104.30 ± 0.52^b^ | 35.60 ± 0.25^b^ |  |
| 3% KMnO_4_/LDPE | 458.04 ± 0.35^d^ | 480.44 ± 0.54^a^ | 497.39 ± 0.56^a^ | 110.79 ± 0.56^b^ | 122.41 ± 2.60^de^ | 110.15 ± 0.11^a^ | 125.08 ± 1.88^f^ | 42.69 ± 0.91^e^ |  |
| 5% KMnO_4_/LDPE | 460.06 ± 1.01^e^ | 479.05 ± 0.98^a^ | 497.13 ± 0.61^a^ | 110.29 ± 0.21^b^ | 118.39 ± 4.19^cd^ | 109.77 ± 0.54^a^ | 120.33 ± 3.01^e^ | 41.07 ± 1.45^d^ |  |
| 1% DA-KMnO_4_/LDPE | 456.36 ± 0.77^d^ | 479.81 ± 1.28^a^ | 497.17 ± 0.22^a^ | 108.12 ± 0.08^a^ | 111.21 ± 0.79^bc^ | 109.95 ± 0.67^a^ | 107.74 ± 0.53^c^ | 36.77 ± 0.26^bc^ |  |
| 3% DA-KMnO_4_/LDPE | 458.80 ± 0.58^d^ | 480.60 ± 1.27^a^ | 496.44 ± 0.25^a^ | 110.81 ± 0.57^b^ | 112.01 ± 2.45^c^ | 110.12 ± 0.79^a^ | 108.26 ± 1.67^cd^ | 38.09 ± 0.80^c^ |  |
| 5% DA-KMnO_4_/LDPE | 461.50 ± 0.59^f^ | 480.89 ± 1.69^a^ | 497.77 ± 0.90^a^ | 110.13 ± 0.09^b^ | 105.57 ± 3.93^a^ | 110.16 ± 0.07^a^ | 103.98 ± 2.72^b^ | 37.19 ± 1.31^bc^ |  |
| 1% Alk-DA-KMnO_4_/LDPE | 456.65 ± 0.09^c^ | 478.64 ± 7.68^a^ | 496.83 ± 0.50^a^ | 110.33 ± 0.23^b^ | 106.73 ± 0.75^a^ | 110.83 ± 0.59^a^ | 107.20 ± 0.54^c^ | 36.59 ± 0.26^bc^ |  |
| 3% Alk-DA-KMnO_4_/LDPE | 458.06 ± 0.04^d^ | 479.80 ± 0.68^a^ | 496.77 ± 0.14^a^ | 108.32 ± 0.23^a^ | 108.73 ± 2.31^a^ | 109.98 ± 0.69^a^ | 112.80 ± 1.70^cd^ | 38.50 ± 0.82^c^ |  |
| 5% Alk-DA-KMnO_4_/LDPE | 461.56 ± 0.62^f^ | 480.81 ± 0.6^a^ | 497.82 ± 0.23^a^ | 109.64 ± 0.45^ab^ | 109.59 ± 3.87^a^ | 109.80 ± 0.57^a^ | 110.15 ± 2.75^cd^ | 37.59 ± 1.33^bc^ |  |

Data provided Mean ± standard deviation (n=2) with the superscript in the same column indicated the significantly differences (p < 0.05).

**Table S 3.** BET Surface area and pore volume of DA and Alk-DA

| Samples | BET Surface area (m^2^/g) | Pore Volume (cm^3^/g) |
| --- | --- | --- |
| DA | 2.223 ± 0.039^a^ | 0.006 ± 0.0001^a^ |
| Alk-DA | 252.542 ± 48.321^b^ | 0.254 ± 0.0499^b^ |

Data provided Mean ± standard deviation (n=2) with the superscript in the same column indicated the significantly differences (p < 0.05).

**Table S 4.** Gas barrier properties of LDPE, 1% DA/LDPE, and 1% Alk-DA-KMnO_4_/LDPE films.

| **Samples** | **OTR 23 °C and 0% RH (cm^3^/m^2^/day)** | **WVTR 38 °C and 90% RH (g/m^2^/day)** |
| --- | --- | --- |
| LDPE | 5694.50 ∓ 222.74^a^ | 11.9395 ∓ 0.38^a^ |
| 1% DA/LDPE | 13389.00 ∓ 797.62^b^ | 13.9595 ∓ 0.23_a_ |
| 1%Alk-DA-KMnO_4_/LDPE | 5946.00 ∓ 91.92^a^ | 12.9440 ∓ 0.08^a^ |

Data provided Mean ± standard deviation (n=2) with the superscript in the same column indicated the significantly differences (p < 0.05).
